# Anthrax infection inhibits the AKT signaling involved in the E-cadherin-mediated adhesion of lung epithelial cells

**DOI:** 10.1111/j.1574-695X.2009.00558.x

**Published:** 2009-07

**Authors:** Taissia Popova, Virginia Espina, Charles Bailey, Lance Liotta, Emanuel Petricoin, Serguei Popov

**Affiliations:** 1 National Center for Biodefense and Infectious Diseases, George Mason UniversityManassas, VA, USA; 2 Center for Applied Proteomics and Molecular Medicine, George Mason UniversityManassas, VA, USA

**Keywords:** reverse-phase microarray, cell signaling, epithelial cells, AKT, cell–cell adherence

## Abstract

The effect of anthrax infection on phosphoprotein signaling was studied in human small airway lung epithelial cells exposed to *B. anthracis* spores of the plasmidless dSterne strain in comparison with the Sterne strain containing the toxigenic plasmid (pXO1). The differential regulation of phosphorylation was found in the mitogen-activated protein kinase cascade (ERK, p38, and P90RSK), the PI3K cascade (AKT, GSK-3α/β), and downstream in the case of the proapoptotic BAD and the transcription factor STAT3. Both strains stimulate phosphorylation of CREB and inhibit phosphorylation of 4E-BP1 required for activation of cap-dependent translation. Downregulation of the survival AKT phosphorylation by the Sterne strain inhibits the process of Ca^2+^-dependent homophilic interaction of E-cadherin (EC) upon formation or repair of cell–cell contacts. Both lethal and edema toxins produced by the Sterne strain inhibit the AKT phosphorylation induced during the EC-mediated signaling. Activity of ERK1/2 and p38 inhibitors indicates that inhibition of AKT phosphorylation takes place through the ERK1/2-PI3K crosstalk. In Sterne spore-challenged mice, a specific inhibitor of PI3K/AKT, wortmannin, accelerates the lethal outcome, and reduction of AKT phosphorylation in the circulating blood cells coincides with the death of animals. We conclude that the PI3K/AKT pathway controlling the integrity of epithelium plays an important survival role in anthrax infection.

## Introduction

Anthrax is a life-threatening disease affecting humans and many species of animals. Major routes of infection are through inhalation, cutaneous abrasions, or ingestion of *Bacillus anthracis* spores. The virulence of *B. anthracis* is mainly attributed to its lethal and edema toxins (LeTx and EdTx, correspondingly) encoded by the XO1 plasmid and the antiphagocytic capsule encoded by the XO2 plasmid. LeTx is a proteolytic inhibitor of mitogen-activated protein kinases (MAPKKs), while EdTx is a bacterial adenylate cyclase generating increased levels of intracellular cyclic AMP (cAMP) (Moayeri & Leppla, 2004). Several lines of evidence suggest that the lethal outcome of anthrax infection may result from the effects of *B. anthracis* toxins and other pathogenic factors on host cell viability (Popov *et al.*, 2002; Moayeri *et al.*, 2003; Kirby, 2004; Moayeri & Leppla, 2004; Firoved *et al.*, 2005) as well as from perturbation of host homeostasis leading to systemic pathophysiological responses such as liver and heart failure, hemorrhage, vascular collapse, and pulmonary edema (Cui *et al.*, 2004, 2007; Popov *et al.*, 2004, 2005; Chung *et al.*, 2006; Watson *et al.*, 2007; Kuo *et al.*, 2008). In connection with the latter, previous reports identified the effects of LeTx, EdTx, proteases of broad specificity, and phospholipases on the barrier function of epithelial and vascular cells (Warfel *et al.*, 2005; Popova *et al.*, 2006). However, the mechanisms by which *B. anthracis* pathogenic factors influence host response in cells of different origins and the relative contributions of these mechanisms to the outcome of infection in patients and experimental animals are not fully understood. Because signal transduction plays a central role in cellular biology and host response mechanisms, we chose to explore the impact of *B. anthracis* infection on innate phosphoprotein signaling pathways in primary human small airway epithelial cells (HSAECs) while taking into account the critical role of lung function in the outcome of inhalation anthrax (Grinberg *et al.*, 2001; Plotkin *et al.*, 2002). While genomic and proteomic studies provide information on the transcriptional and translational changes induced by activating cell signaling pathways, the activities of nearly all signaling proteins are modulated by their post-translational phosphorylations. We expected that information gleaned in the study would help identify pathogenic host responses for further evaluation of their contribution to the lethal outcome of infection. We compared the levels of phosphorylated signaling proteins generated in the cells in response to the bacteria of the toxigenic, nonencapsulated Sterne strain (lethal in mice) and the isogenic nontoxigenic, nonencapsulated delta Sterne (dSterne) strain (nonlethal in mice), and identified differences correlating with strain virulence. Among these, our attention was attracted to the inhibition of the PI3K/AKT signaling pathway and the role of anthrax toxins in this process.

AKT (protein kinase B) is an established pluripotent key mediator of cell survival after various insults, including growth factor withdrawal, chemical and physical stress, treatment with therapeutic agents, and ischemic shock (Datta *et al.*, 1999; Brazil *et al.*, 2002; Franke *et al.*, 2003; Peng *et al.*, 2003). However, its role in anthrax pathogenesis has just started to emerge (Tucker *et al.*, 2003; Comer *et al.*, 2005a, b; Ha *et al.*, 2007). AKT is mediated by a variety of stimuli at the intersection of multiple signaling pathways and therefore displays an extremely complex behavior. Three isoforms of AKT are major targets of class IA PI3-kinase, which enhances the level of lipid second messenger PI-3,4,5-triphosphate upon stimulation, leading to its binding to the pleckstrin homology domain of AKT (Datta *et al.*, 1999; Brazil *et al.*, 2002). AKT is activated by PI3K in response to growth, and survival factors through phosphorylation of T308 and S473. Murine knock-outs of the PI3K genes result in embryonic lethality, and double AKT-1 and -2 knock-out animals die within a few hours of birth from respiratory failure (Peng *et al.*, 2003), indicating a critical role of PI3K/AKT signaling in lung function and tissue homeostasis. AKT-dependent protein phosphorylation directly inhibits the activities of several proapoptotic proteins and promotes cell survival through indirect effects on p53 and NF-κB and via stimulation of glucose metabolism and protein synthesis. AKT also plays a protective role directed at eliminating nonpathogenic bacteria (Datta *et al.*, 1999; Brazil *et al.*, 2002; Wesche *et al.*, 2005). The class I PI3K-AKT axis has key roles in the transduction of survival and/or mitogenic signals from receptor tyrosine kinases and cell adhesion events, including those mediated by the calcium-dependent E-cadherin (EC), the major cell–cell adhesion protein of adherens junctions (Niessen & Gottardi, 2008). Class 1A PI3-kinase is recruited to EC-based contacts as epithelial cells form adherens junctions (Pece *et al.*, 1999; Pece & Gutkind, 2000; Pang *et al.*, 2005). Such EC-activated PI3-kinase signaling is necessary for both adhesive strengthening and the efficient assembly of EC adhesive contacts (Pece *et al.*, 1999; Pang *et al.*, 2005; Perrais *et al.*, 2007; Perez *et al.*, 2008).

In this report we demonstrate a broad impact of *B. anthracis* infection on host cell phosphoprotein signaling in infected HSAECs, including inhibition of the PI3K/AKT pathway. We also show that this pathway is causally important for the survival of *B. anthracis* spore-challenged mice. LeTx and EdTx contribute to the inhibition of AKT phosphorylation and thus interfere with the signaling required for the assembly of the EC-mediated adherens junctions.

## Materials and methods

### Reagents and antibodies

Cell culture reagents were from CellGro (Herndon, VA). Antibodies against total and phosphorylated forms of the following proteins used for reverse-phase protein microarrays (RPMA) and Western blots were from Cell Signaling Technology (Beverly, MA) and were used at the dilutions indicated: 1 : 20 for p70 S6 kinase (Thr389); 1 : 50 for c-Abl (Thr 735), Stat5 (Tyr694), 4E-BP1 (Ser65); 1 : 100 for AKT (Ser473), MEK1/2 (Ser 217/221), pIKBa (Ser32/Ser36), Bad (Ser112, 136, 155), 4E-BP1 (Thr70), GSK-3α/β (Ser21/9), CREB (Ser 133), Stat3 (Ser727, Tyr705), Jak1 (Tyr1022/1023), FAK (Tyr576/577), Etk (Tyr 40), Elk-1 (Ser383), MARCKS (Ser152/156); 1 : 200 for mTOR (Ser2448), eNOS (Ser1177), Pyk2 (Tyr402), FADD (Ser194), Stat6 (Tyr641), Bcl-2 (Ser70); 1 : 250 for p38 (Thr180/Tyr182), IL-1β-cleaved (Asp116); 1 : 400 for p90RSK (Ser380); 1 : 500 for PKC-δ (Thr505), PKC-α/β (Thr638/641), PKC-θ (Thr538), caspase-7 cleaved (Asp198), caspase-9 cleaved (Asp330), caspase-3 cleaved (Asp175), ERK 1/2 (Thr202/Tyr204), pPKC-z (Thr410/403), Src (Tyr527), Stat1 (Tyr701), Bax; 1 : 1000 for actin, 4E-BP1 (Thr37/46), EC, Bcl-xL; 1 : 2000 for eIF4G (Ser1108). Recombinant protective antigen, lethal factor, and edema factor were from List Biological Laboratories (Campbell, CA). Other reagents were from Sigma-Aldrich (St. Louis, MO).

### Challenge of lung epithelial cells with spores and supernatants of bacterial cultures

HSAECs (Cambrex Inc., Walkersville, MD) from two different donors were grown according to the vendor's protocol in Ham's F12 medium supplemented with nonessential amino acids, pyruvate, β-mercaptoethanol and 10% fetal calf serum (FCS) at 37 °C in an atmosphere of 5% CO_2_. The cells were adapted to these culture conditions during four passages and then were used for the preparation of the frozen stock. Further experiments were performed with cells at passages between 5 and 10. For microarray experiments, confluent HSAECs from one of the donors (10^6^ per well in 12-well plates) were incubated in Complete Serum-Free Medium® (CellGro) plus 1% FCS for 16 h before challenge with *B. anthracis* spores at the multiplicity of infection (MOI) 1 and 10. According to the manufacturer this medium is a proprietary serum-free and low-protein formulation based on DMEM/F12, RPMI 1640, and McCoy's 5A that does not contain any insulin, transferrin, cholesterol, growth or attachment factors. A mixture of trace elements and high molecular weight carbohydrates, extra vitamins, a nonanimal protein source, and a small amount of high quality bovine serum albumin (1 g L^−1^) are added to support growth and viability over long-term passages of hybridomas, suspension, and adherent cultures. The toxigenic *B. anthracis* Sterne strain 43F2 (pXO1^+^, pXO2^−^) and the nontoxigenic, plasmidless strain dSterne (pXO1^−^, pXO2^−^) were from Colorado Serum Company (CO) and the National Center for Biodefense and Infectious Diseases, George Mason University (VA), correspondingly. The absence of pXO1 in the dSterne strain was confirmed by PCR with the protective antigen-specific primers. Both strains have identical growth curves in the culture medium used for challenge experiments (Supporting Information, Fig. S1). The spores were prepared as described in Popov *et al.* (2002). At certain time points post-HSAEC spore challenge, supernatant was removed and cells were lysed and immediately boiled for 10 min in 100 μL of 1 : 1 mixture of T-PER Reagent (Pierce, IL) and 2 × Tris-glycine sodium dodecyl sulfate (SDS) sample buffer (Novex, Invitrogen, CA) in the presence of 2.5%β-mercaptoethanol, and protease and phosphatase inhibitors (Pierce). Lysed samples were stored at −80 °C. Three independent cell challenge experiments were performed for the microarray and Western blot analyses.

The viability of cells after exposure to spores was determined using the Alamar Blue (Sigma-Aldrich). Alamar Blue is a water-soluble, nontoxic, fluorometric/colorimetric growth indicator. Cellular growth and metabolism reduce the dye and cause a color change to red. The cells grown in 96-well plates were incubated with Alamar Blue in the presence of antibiotics (100 μg mL^−1^ of each penicillin and streptomycin) added to the samples 1 h before assay to inhibit the metabolic influence of bacterial vegetative cells. Fluorescence of the dye was measured at 590 nm after excitation at 530 nm. In the negative control experiments, bacteria grown without HSAECs did not demonstrate Alamar Blue fluorescence after incubation with antibiotics.

In the experiments with bacterial culture supernatants (SUPs), confluent HSAECs were seeded into the 12- or 48-well culture plates, cultivated as described above, washed three times with Hanks balanced salt solution (HBSS), and incubated with SUPs at 37 °C, 5% CO_2_. SUPs were produced by inoculation of spore suspension to a final concentration of 6 × 10^6^ spores mL^−1^ into Complete Serum-Free Medium® and incubation without agitation at 37 °C, 5% CO_2_. Bacteria were removed by centrifugation, and SUPs were supplemented with 100 μg mL^−1^ of each streptomycin and penicillin to exclude growth of any contaminating bacteria upon incubation with HSAECs. The early apoptotic changes in exposed cells were detected with APOAC-1KT kit (Sigma-Aldrich) according to manufacturer's protocol using fluorescence microscope Nikon Eclipse 90i with C1SHS camera. Briefly, after incubation with SUPs, cells grown on slides were washed 3 times with the binding buffer. Annexin V conjugated with Cyt3.18 dye was added for 10 min, the cells were washed five times with the binding buffer, and treated with fixation/permeabilization solution (BD Biosciences, MA) for 20 min. Finally, the slides were washed three times with phosphate-buffered saline (PBS), dried at room temperature, and mounted using antifade reagent Prolong Gold (Invitrogen) containing a nuclear DAPI stain.

For immunofluorescent staining with anti-EC antibody, HSAECs were seeded (6 × 10^4^ per well of eight-chamber plate from BD Biosciences), grown in 0.5 mL well^−1^ of Ham's F12 medium with 10% FCS at 37 °C and 5% CO_2_ for 7 days, and treated with SUPs of Sterne and dSterne strains for 4 h. After treatment the cells were briefly washed with Complete Serum-Free Medium®, fixed with 3.2% paraformaldehyde in HBSS with Ca^2+^ and Mg^2+^ for 10 min, washed twice with the blocking buffer (10% normal goat serum from Jackson ImmunoResearch Laboratories, PA, in HBSS), permeabilized with cold 1% Triton X-100 in the blocking buffer for 10 min, washed three times with the blocking buffer, and finally incubated in the blocking buffer for 30 min. The cells were then incubated with the first antibody (rabbit anti-EC, H-108 from Santa Cruz, CA) in blocking buffer (2 μg mL^−1^) at 4 °C overnight, washed three times in the washing buffer (150 mM NaCl, 25 mM Tris, pH 7.5, 0.05% Tween 20), incubated with 2 μg mL^−1^ of secondary antibody in the blocking buffer (goat anti-rabbit conjugate with Alexa 488 from Cell Signaling Technology, CA) for 30 min, washed three times in the washing buffer, and finally mounted as described above.

For Western blot analyses, the supernatants of the exposed HSAECs were quickly discarded and the cells were lysed with 50 μL per well of 1 × SDS-polyacrylamide gel electrophoresis (PAGE) loading buffer with cocktail of protease inhibitors (Pierce), phosphatase inhibitors (50 mM sodium fluoride, 0.2 mM sodium vanadate), 2 mM EDTA, and 6 mM dithiothreitol.

### RPMA and Western blot analyses

Three nanoliters of each sample were arrayed by direct contact printing using a high-resolution 2470 arrayer (Aushon Biosystems, Billerica, MA) onto nitrocellulose slides (Whatman, MA). Samples were printed as duplicates of the five-point serial dilution curves to ensure the linear detection range for the antibody concentrations used. Slides were kept at −20 °C before analysis with antibodies. To estimate the total protein amount, selected slides were stained with Sypro Ruby Protein Blot Stain (Molecular Probes, Eugene, OR) and visualized on a Fluorchem imaging system (Alpha Innotech, San Leandro, CA). Slides were stained with specific antibodies on an automated slide stainer (Dako Cytomation, Carpinteria, CA) using a biotin-linked peroxidase-catalyzed signal amplification. The arrayed slides were placed into 1 × Re-Blot solution (Chemicon, Temecula, CA) for 15 min, washed two times for 5 min each in PBS, placed into I-Block solution (Applied Biosystems, Foster City, CA) in PBS/0.1% Tween-20 for at least 2 h, and then immunostained using an automatic slide stainer (Autostainer, Dako Cytomation) using manufacturer-supplied reagents. Briefly, the slides were incubated for 5 min with hydrogen peroxide, rinsed with high-salt Tris-buffered saline (CSA Buffer, Dako) supplemented with 0.1% Tween-20, blocked with avidin block solution for 10 min, rinsed with CSA buffer, and then incubated with biotin block solution for 10 min. After another CSA buffer rinse, 5-min incubation with protein block solution was followed by air drying. The slides were then incubated with either a specific primary antibody diluted in Dako Antibody Diluent or, as a control, with only DAKO Antibody Diluent for 30 min. Before use on the RPMA, every antibody underwent extensive validation for specificity (e.g. single band on Western blot, peptide competition, ligand induction for phosphospecific reagents). The slides were then washed with CSA buffer and incubated with a secondary biotinylated goat anti-rabbit immunoglobulin G H+L antibody (1 : 5000) (Vector Labs, Burlingame, CA) for 15 min. For amplification purposes, the slides were washed with CSA buffer and incubated with streptavidin–horseradish peroxidase for 15 min, followed by a CSA buffer rinse. Slides were then incubated in diaminobenzidine chromogen diluted in Dako diaminobenzidine diluent for 5 min, washed in deionized water and imaged using a UMAX PowerLook III scanner (UMAX, Dallas, TX) at 600 dpi. The images were analyzed with software alphaease fc (Alpha Innotech). For each antibody, the average pixel intensity value for negative control (staining with only second antibody) was subtracted from the average pixel intensity value for specific antibody and then divided by the corresponding value of the Sypro-stained total protein slide. For RPMA, each sample dilution curve in each experiment was fitted with a nonlinear approximation equation and statistically evaluated using the software prizm (Graphpad Software, CA). The dilutions of different samples, which resulted in the same signal intensity arbitrarily chosen within the linear response interval, were used to calculate relative analyte concentrations and their confidence intervals (*P*=0.05). The microarray experiments were repeated to ensure consistent results. HSAECs from two different donors demonstrated similar results (not shown). Signaling proteins demonstrating difference between control and challenged cells were further tested with Western blots using 20 μL of cell lysates, 1 : 1000-diluted primary antibodies, and 1 : 7500-diluted secondary antibody. Proteins were transferred to nitrocellulose membrane using iBlot Gel Transfer Device (Invitrogen). The membranes were developed with SuperSignal West Femto Maximum Sensitivity Substrate (Pierce), and band intensities were measured with a Molecular Imager ChemiDoc XRS System (Bio-Rad, CA). The intensities of bands were calculated relative to untreated controls after densitometry using the quantity one 4.6.5 program (Bio-Rad). All measurements were performed in triplicate.

### Animal experiments

Male 6–8-week-old DBA/2 mice (Jackson Labs) received food and water *ad libitum* and were challenged with *B. anthracis* Sterne spores [1 × 10^6^ spores, intraperitoneally (i.p.) five animals per challenge group] on day 0. Survival of animals was monitored for 10 days. Wortmannin (Sigma-Aldrich) was administered i.p. 1 h before the spore challenge, and then once daily until death. To obtain blood samples, the challenged animals were bled from the orbital sinus vein and then immediately sacrificed by cervical dislocation. Blood was mixed with equal volume of 2 × SDS-PAGE loading buffer containing 12 mM dithiothreitol, 2 × cocktail of protease inhibitors (Pierce), phosphatase inhibitors (100 mM sodium fluoride, 0.4 mM sodium vanadate), and 4 mM EDTA. Samples were boiled for 10 min and used for Western blot with pAKT-specific antibody as described above. Densitometry was performed and relative intensities of bands were calculated. Analysis was carried out in triplicate. The protocol of experiments was approved by the Institutional Animal Care and Use Committee of George Mason University, Manassas, VA.

### Statistical analyses

All data are presented as mean±confidence interval with a significance level of 0.95 calculated using the SDs of the measurements. The Kaplan–Meier log-rank statistical test was applied to evaluate reliability of survival data.

## Results

### Phosphoproteomic analysis reveals differential innate responses of HSAECs to the lethal and nonlethal *B. anthracis* strains

**Fig. 1:**
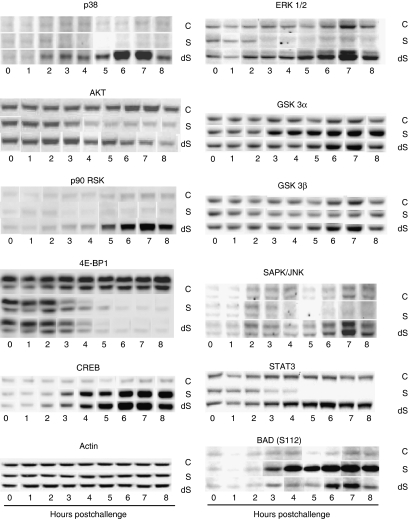
Dynamics of signaling protein phosphorylation. HSAECs were exposed to *Bacillus anthracis* spores of the nontoxigenic dSterne strain (dS) or the toxigenic Sterne strain (S) at MOI 10. The cells were lysed, and the lysates were used for Western blots using primary antibodies against phosphorylated forms of proteins as described in Materials and methods. Intensities of actin bands served as a gel-loading control. Data are representative of three independent challenge experiments. Densitometry of Western blot data for AKT and GSK-α/β is shown in Fig. S2. C, control.

**Fig. 2:**
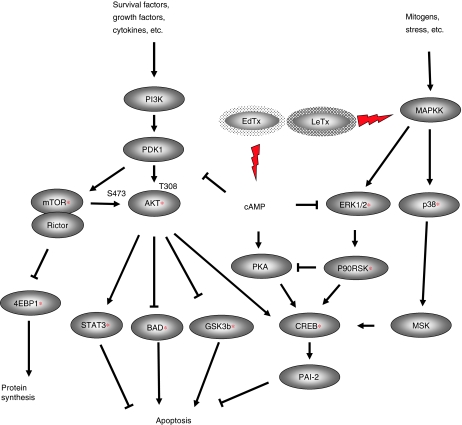
Fragments of MAPKK and PI3K prosurvival pathways in HSAECs exposed to *Bacillus anthracis*. Asterisks indicate differential phosphorylation detected by the microarray.

To study the dynamics of host cell signaling phosphorylation during *B. anthracis* infection, we used a model of HSAECs challenged with *B. anthracis* spores, which germinated and grew as vegetative bacteria atop of the host cells. We suggested that this model would be instructive regarding the initial interaction of lung epithelium with inhaled spores, as well as the late septicemic stage of infection characterized by dissemination of vegetative bacteria and their secreted products to the lungs and other epithelium-reach organs. Cell lysates were prepared at different times postchallenge to encompass the period of infection from 10 min to 8 h at MOI 1 and 10. For initial identification of signaling proteins the lysates were used for fabrication of RPMA on nitrocellulose membrane slides. Each slide was then probed with one of 40 different antibodies specific against phosphorylated forms of signaling proteins (see Materials and methods) selected to monitor the molecular networks involved in host responses most likely affected by bacterial exposure, namely survival, apoptosis, inflammation, growth, differentiation, and immune response. This microarray technique was extensively validated in our previous studies (Wulfkuhle *et al.*, 2006) with regard to specificity of antibodies, sensitivity, and accuracy of phosphoprotein detection in cell lysates. Of the 40 different signaling proteins, the relative levels of 20 phosphorylated proteins showed statistically significant (*P*<0.05) difference from those in the control untreated cells at one or several time points upon exposure to either toxigenic or nontoxigenic *B. anthracis* strain (Table S1). The responses detected at both MOIs were similar but stronger at MOI 10 (not shown). To determine whether the detected phosphorylation events reflect a biologically cohesive set of signaling processes, the data were evaluated using the software package pathwayarchitect 2.0 and the associated database of biological interactions (Stratagene, CA). All proteins whose phosphorylations were significantly changed during the infection in HSAECs were found to belong to a single direct-interaction network (*P*<10^−5^) where all signaling nodes are interconnected, and no irrelevant nodes without direct connections are present. The high functional importance of these network proteins is reflected in the connectivity index measuring the number of interactions with other proteins in the database (Table S1). Eleven of the above proteins were chosen for further Western blot analyses as time course curves for MOI 10 (Fig. 1). A part of the signaling network relevant to these proteins is illustrated in Fig. 2.

All of the signaling detected represents transient innate responses of viable cells within 1–8 h postchallenge as detected by the Alamar Blue assay (see Materials and methods). Among the key host cell signaling proteins whose phosphorylation was differentially inhibited by the toxigenic Sterne strain and stimulated by the dSterne strain are the stress-activated kinase p38, ERK1/2 (p44/42 MAPK), and its downstream target, p90 RSK, all members of the prosurvival MAPKs pathway. Consistent with these observations, MAPKKs 1/2 and 3/6 regulating the downstream kinases ERK and p38 are well-known specific targets of LeTx proteolytic activity (Duesbery & Vande Woude, 1999; Pellizzari *et al.*, 2000). Their inhibition by LeTx leads to a reduction in proinflammatory responses and the induction of apoptosis in macrophages and endothelial cells (Popov *et al.*, 2002; Kirby, 2004). In addition to the effect on the host cell MAPK pathway, we detected a significant reduction in phosphorylation within the PI3K/AKT prosurvival signaling pathway in the presence of pXO1-positive bacteria, in comparison with the pXO1-negative ones. Specifically, the HSAECs showed reduced phosphorylation of the global regulator of survival pathways serine/threonine kinase AKT (protein kinase B) at its major activating site S473, and its downstream target, the proapoptotic glycogen synthase kinase 3β (GSK-3β) (Fig. 1b). However, the phosphorylation of the functionally redundant GSK-3α was stimulated by both bacterial strains. Differential stimulation/inhibition of phosphorylation was also found in the case of signal transducer and activator of transcription, STAT3, and its regulator, Janus tyrosine kinase 1 (JAK1). Further, the transcriptional factor CREB showed a strong upregulation by both strains, while the phosphorylation of translation inhibitor 4E-BP1 required for activation of the cap-dependent translation was reduced by both strains indicating a contribution of the pXO1-independent pathogenic factors. In contrast to our expectations, the proapoptotic BCL-2-family member BAD was strongly phosphorylated at its inactivation site S112 by the Sterne strain. Similar but smaller effect was seen in HSAECs exposed to the pXO1-negative strain. Overall, the results of our initial analysis identified a number of features of the HSAEC signaling responses potentially relevant to the virulence of the pXO1-positive *B. anthracis* Sterne. In further analyses, we focused our study on the novel role of AKT in the host response to *B. anthracis* infection and explored the causal significance of this host cell pathway modulation in the pathogenic process.

### Soluble factors released by *B. anthracis* into culture medium induce apoptosis in HSAECs and inhibit transient activation of the PI3K/AKT pathway associated with remodeling of EC in adherens junctions

In order to reduce the complexity of our experimental model, we tested whether the effect of *B. anthracis* infection on host cell signaling could be mimicked by the soluble factors secreted by bacterial cells. For this purpose, we grew *B. anthracis* cultures in the conditions used for the RPMA experiments and tested the supernatants of these cultures (SUPs), as well as the pelleted and washed bacteria harvested at different stages of growth (a midlog phase at 4 h, and a late-log phase at 8 h) for their effects on cell viability, induction of apoptosis, and inhibition of AKT phosphorylation.

**Fig. 3:**
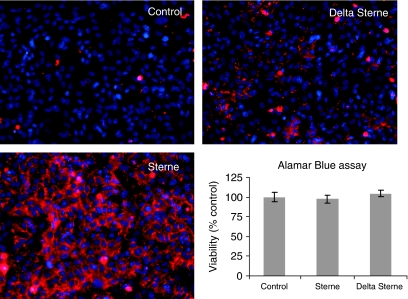
HSAECs treated with SUPs reveals early apoptotic changes in plasma membrane. Confluent HSAECs were treated with 8-h SUPs of Sterne and dSterne strains for 2 h, stained with Annexin V conjugated with Cy-3.18 (red color), washed, and observed under fluorescent microscope (× 200). Blue color corresponds to DAPI-stained nuclei. Bar graph shows the results of the Alamar Blue assay with 1x10^5^ cells/well. Error bars correspond to 95% confidence intervals (*n*=3).

Freshly confluent HSAECs incubated with SUPs for 2 h demonstrated no detectable loss of HSAECs' viability compared with control cells in the assays with Alamar Blue (Fig. 3) and carboxyfluorescein diacetate, which enters the cell and is hydrolyzed by the esterases present in living cells to the fluorescent compound 6-carboxyfluorescein (not shown). However, HSAECs treated with the 8-h SUP of the Sterne strain, in comparison with the 8-h SUP of the dSterne strain and untreated cells, showed massive fluorescence after staining with Cy-3.18-conjugated Annexin V specific for early apoptotic changes in the plasma membrane (Fig. 3). These observations are in line with our previous observations on the proapoptotic signaling by the Sterne strain (Popov *et al.*, 2002).

**Fig. 4:**
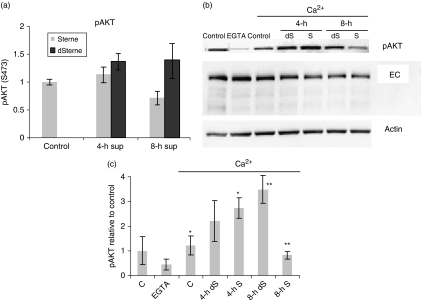
Analyses of pAKT and EC in HSAECs treated with SUPs directly (a) or in the conditions of calcium switch (b, c). HSAECs grown for 5 days after reaching confluence were washed three times with 0.5 mL of prewarmed HBSS with Ca^2+^ and Mg^2+^ and treated in triplicate with the indicated SUPs for 2 h. After incubation, the SUPs were removed and the cells were either (a) lysed for Western blot analysis, or (b, c) treated with 0.5 mL of 4 mM EGTA in DMEM/F-12 medium. After 30 min, EGTA was removed and replaced with DMEM/F-12 medium. After 15 min, the medium was removed and the cells were lysed in 50 μL of SDS-PAGE loading buffer. Samples were run in 4–12% Bis–Tris gel for Western blot with pAKT- and EC-specific antibodies as described in Materials and methods. The intensities of bands in western blot were calculated relative to untreated cells after densitometry. (a, c) Densitometry results of three independent experiments made in triplicate. (b) Image of a typical Western blot. Error bars represent 95% confidence intervals of means; asterisks indicate ^*^P<0.01, ^**^P<0.01.

The differential effect between Sterne and dSterne strains on AKT (S473) phosphorylation was detected using the 8-h SUPs after 2-h incubation (Fig. 4a). The washed bacterial cell pellets resuspended in the culture medium (supplemented with 100 μg mL^−1^ of both penicillin and streptomycin to prevent bacterial growth) did not affect AKT phosphorylation (not shown). In order to explore the mechanism of differential AKT phosphorylation, we suggested that it might be relevant to changes in the intercellular contacts known as sites of intense phospho-tyrosine signaling (Lu *et al.*, 2001). EC is known to mediate cell–cell adhesion through Ca^2+^-dependent homophilic interaction of its extracellular domain. Removal of extracellular calcium ions (e.g. by addition of metal chelators such as EGTA) disrupts this interaction and causes internalization of EC followed by its ubiquitination and degradation (Shen *et al.*, 2008). Loss of EC-mediated adhesion is associated with a cascade of intracellular signaling, including inhibition of PI3-kinase-dependent AKT phosphorylation at S473. Addition of Ca^2+^ restores EC engagement, followed by a rapid transient activation of AKT and subsequent maturation of cell–cell contacts independently from other interactions; however, the process of adhesive strengthening during maturation is not accompanied by obvious phenotypical changes (Pece *et al.*, 1999; Pece & Gutkind, 2000; Lu *et al.*, 2001; Pang *et al.*, 2005; Perrais *et al.*, 2007; Perez *et al.*, 2008). Therefore, the ‘calcium switch’ technique can be used as a selective means to assess the functional condition of EC on the cell surface by monitoring the AKT phosphorylation during adherens junctions' dissociation and reassociation. A synchronous initiation of EC reassociation after addition of calcium increases sensitivity of the assay. We tested HSAECs after pretreatment with SUPs in the conditions of calcium switch. Restoration of Ca^2+^ in the control untreated cells and the cells treated with SUPs generated increased amounts of pAKT (Fig. 4b and c). However, the 8-h SUP of the toxigenic Sterne strain inhibited the EC-mediated pAKT response to the level of untreated cells.

**Fig. 5:**
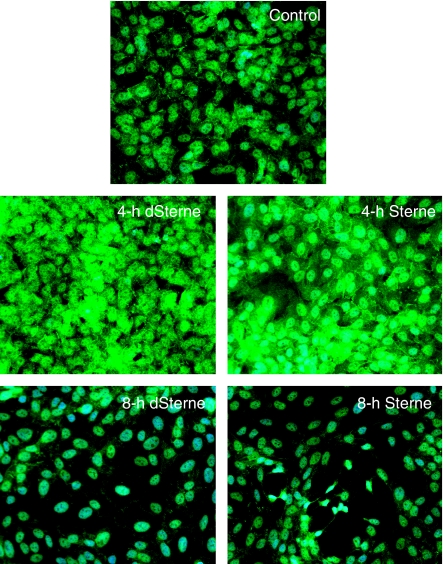
Immunofluorescence staining of EC in HSAECs treated with SUPs reveals changes in the amount and localization of EC. Confluent HSAECs were treated with SUPs of Sterne and dSterne strains for 4 h and stained with anti-EC antibody, followed by Alexa 488-conjugated secondary antibody. Green color corresponds to EC and blue to DAPI-stained nuclei. Images represent characteristic features of cells from three different slides corresponding to a particular treatment condition.

We previously reported that several *B. anthracis* proteins, including LeTx, changed a pattern of EC staining at the cell–cell contacts of cultured murine mammary gland epithelial cells (Popova *et al.*, 2006). In the case of confluent HSAEC monolayers, the immunofluorescent staining of untreated cells using H-108 antibody specific for the extracellular domain of this protein demonstrated a peripheral EC staining along with a large amount of EC diffusely distributed in the cell cytoplasm (Fig. 5). Treatment of HSAECs with 4-h SUPs of both bacterial strains did not result in obvious changes in cell morphology, although in the case of 8-h dSterne SUPs the monolayers displayed the regions containing hypertrophic cells with increased size and ‘stretched’ cytoplasm. However, these cells still maintained cell–cell contacts. Treatment of HSAECs with 8-h Sterne SUPs resulted in the appearance of intercellular gaps formed by the cells with a collapsed cytoplasm. Taken together, these results demonstrated that secreted factors of *B. anthracis* modulate the PI3K/AKT pathway signaling induced in the process of Ca^2+^-dependent homophilic interaction of EC and thereby interfere with the EC-mediated cell–cell adhesion in the adherens junctions. Upregulation of AKT phosphorylation at the early stages of infection (Fig. 1b) might represent a compensatory response to strengthen the cell contacts damaged by yet unknown mechanism. Downregulation of this response by the Sterne strain is expected to abrogate the reparation process.

### Anthrax toxins inhibit AKT phosphorylation induced by homophilic association of EC in the presence of Ca^2+^

**Fig. 6:**
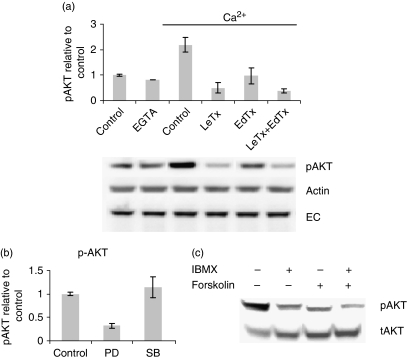
Effect of LeTx and EdTx on phosphorylation of AKT in calcium switch conditions. HSAECs were seeded, grown, and tested as described in Fig. 4 in conditions of calcium switch (a, b) or serum stimulation (c) after pretreatment with toxins or specific inhibitors. (a) Pretreatment with toxins. Toxins (1 μg mL^−1^ of lethal factor or edema factor plus 3 μg mL^−1^ of protective antigen) were dissolved in Complete Serum-Free Medium® and incubated with cells for 1 h before calcium switch. Toxins were removed and 4 mM EGTA containing the same concentration of toxins as above in DMEM/F-12 medium (without Ca^2+^ and Mg^2+^) were added to the cells for 15 min. Four independent Western blot experiments with pAKT-specific antibody were carried out in triplicate with similar results. Actin band detected with a specific antibody served as a loading control. (b) Pretreatment with specific inhibitors. The inhibitors PD 98059 (50 μM) and SB 203580 (10 μM) were dissolved in DMEM/F-12 medium and incubated with cells for 1 h before addition of EGTA as described in (a). Amounts of pERK1/2 and pAKT were determined by Western blot. The experiment was carried out in triplicate and repeated twice. (c) Stimulation of adenylate cyclase. Forskolin (100 μM) and IBMX (100 μM) were dissolved in DMEM/F-12 medium and incubated with cells for 4 h. Serum-free medium was replaced with the one containing 10% FCS, and Western blot for pAKT was performed after 30 min of incubation. Data are representative of two independent experiments.

The observed rapid inhibition of AKT phosphorylation by the pXO1-positive strain suggested a direct role of anthrax toxins in this process. In order to obtain data in support of this suggestion, we first confirmed the expression of toxins in the conditions of our experiments. Our enzyme-linked immunosorbent assay data demonstrated the appearance of protective antigen (the binding subunit of anthrax toxins) in the SUP at 4 h after spore inoculation in the amount of 1 μg mL^−1^. The 8-h SUP contained >3 μg mL^−1^ of protective antigen (not shown). The HSAECs were pretreated with purified LeTx and EdTx for 1 h and tested in the calcium switch experiments. Both toxins individually and in combination with each other were capable of inhibiting AKT phosphorylation to about 20% of its Ca^2+^-induced level in control untreated cells, without a detectable change in the amount of cell-associated EC (Fig. 6a).

In order to confirm that the effect of LeTx was relevant to MAPKK inactivation, we carried out experiments with chemical inhibitors of MAPKKs. In the conditions of calcium switch, PD98059, a specific inhibitor of ERK1/2, reduced AKT phosphorylation similar to LeTx (Fig. 6b). This is consistent with the known function of LeTx as an inhibitor of ERK1/2 and indicates a cross-talk between ERK1/2 and AKT in calcium-mediated EC signaling. However, with the p38 pathway inhibitor, SB203580, no statistically reliable change in AKT phosphorylation was observed (Fig. 6b).

Generation of cAMP is the only known function of EdTx. To confirm the existence of a cAMP-AKT pathway, the cells were treated with forskolin, an inducer of cellular adenylate cyclase, in the presence or absence of the phosphodiesterase inhibitor IBMX (which prevents intracellular degradation of cAMP). The level of pAKT after stimulation with serum in culture medium was considerably reduced by this treatment (Fig. 6c). Overall, we concluded that the magnitude of the toxins' effect is consistent with their major role in the differential inhibition of AKT by Sterne strain. However, our experiments do not exclude a potential contribution from other pXO1-encoded proteins.

### Inhibition of PI3K/AKT pathway increases susceptibility of mice to *B. anthracis* infection

**Fig. 7:**
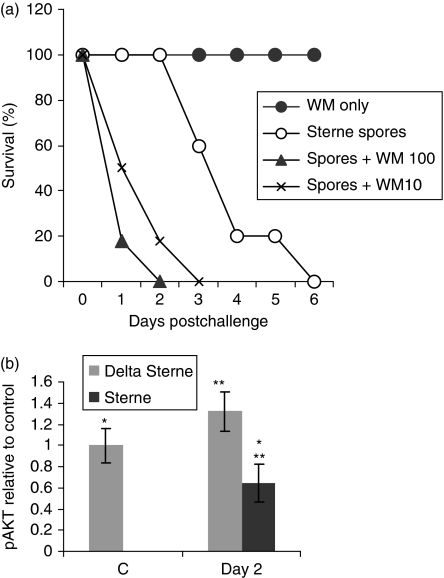
Inhibition of PI3K/AKT pathway correlates with susceptibility of mice to *Bacillus anthracis* infection. (a) Mice (five animals per challenge group) were administered a bolus daily dose of 10 and 100 μg of wortmannin (WM 10 and WM 100, correspondingly), and after 1 h were challenged with Sterne strain spores. Wortmannin administration was continued for two more days. Wortmannin accelerated lethal outcome (*P*<0.001 compared with spore-only challenge). (b) Levels of pAKT were determined using Western blot in circulating blood cells of mice challenged i.p. with spores of Sterne and dSterne strains (^*^P<0.05, ^**^P<0.01, *n*=4 per challenge group).

In order to test the relevance of AKT inhibition to the virulence of *B. anthracis* infection and extend our analysis beyond pure correlative observations, mice were administered an inhibitor of class I PI3K, wortmannin, and then challenged with spores of the toxigenic Sterne strain. (The nontoxigenic strain does not produce a productive infection and therefore could not be tested.) Similar approaches have been used previously to investigate the role of the PI3K pathway in endotoxemic and oxidant-injured mice (Lu *et al.*, 2001; Shen *et al.*, 2008). A control group of mice was challenged with Sterne spores only. A third group received wortmannin only. Figure 7a shows that all mice in the control groups survived, while all mice coadministered the spores and the inhibitor died in an inhibitor dose-dependent manner faster than the animals challenged with spores only. These data indicated that PI3K/AKT signaling plays a protective role, and its inhibition by *B. anthracis* might contribute to *B. anthracis*' overall virulence.

To determine whether differential inhibition of AKT signaling by the two *B. anthracis* strains detected in HSAECs takes place *in vivo* during systemic infection, we challenged mice with a lethal dose of Sterne spores and analyzed the levels of AKT activation in circulating blood cells from mice bled at different stages of infection. In the control group, animals were challenged with the same amount of nonlethal dSterne spores. Figure 7b shows a considerable reduction of pAKT at day 2 post infection (p.i.) in the Sterne-challenged mice, relative to the dSterne-challenged and control mice, similar to the differential response of HSAECs. We have previously shown that day 2 p.i. is typically the onset of the active anthrax infection preceding the death of the animals, which takes place on and after day 3 p.i. (Popov *et al.*, 2005). Consistent with this course of the disease, 50% of the animals died at day 3 p.i. Overall, these results demonstrated that down-modulation of AKT phosphorylation represents an important pathogenic feature of the toxigenic, lethal infection.

## Discussion

Lung epithelial cells are positioned at the foremost interface between the environment and the internal compartments of the body and provide both barrier and signaling functions to protect against infection by airborne pathogens. Therefore, these cells are expected to behave as a robust but sensitive barrier that quickly responds to pathogenic insults. In this report, we characterized the early innate responses of primary lung epithelial cells to the pathogenic factors secreted by *B. anthracis* using an RPMA microarray to broadly measure cell signaling pathway activation and confirmed these findings using Western blots. Although LeTx is known to inhibit the MAPK cascade, the full picture of host cell signaling changes in response to anthrax infection remains largely unknown. The ability to simultaneously detect changes in the phosphorylation of a large number of proteins over a time course in the comparative experiments with the matched pair of isogenic pXO1-positive and pXO1-negative *B. anthracis* strains helped us expand the information available regarding specific signaling pathways altered by the infection. Our results showed that HSAECs responded to the nonlethal *B. anthracis* strain with a cascade of host signaling emanating from major prosurvival MAPK and PI3K/AKT pathways, while infection with the lethal strain caused a reduction in activation/phosphorylation of key members of entire cell survival signaling networks. A number of previous studies did not utilize this comparative approach and typically reported results using only virulent strains or toxins (Tucker *et al.*, 2003; Bergman *et al.*, 2005; Comer *et al.*, 2005a, b, 2006). Consequently, silencing of host responses may not have always been detected and recognized as a pathogenic process.

The fundamental importance of MAPK and AKT in cell behavior led us to explore the correlation between host lung epithelial cell signaling and the most important direct biological outcome of anthrax exposure, mortality. Reduced phosphorylation of AKT, due to its central role in survival, typically follows proapoptotic insults, while the constitutive activation of AKT is able to block cell death induced by a variety of apoptotic stimuli. Both animal and recent patient studies have established lung endothelial and epithelial apoptosis as an important part of the pathogenesis of acute lung injury (Mantell *et al.*, 1999; Albertine *et al.*, 2002). Consistent with this, our experiments with the Annexin V-stained cells revealed early apoptotic changes induced by the toxigenic strain in the plasma membrane. In our animal experiments, wortmannin, a specific inhibitor of PI3K, accelerated death of challenged mice, while the infectious process caused inhibition of AKT phosphorylation in the circulating blood cells. These data suggest that the conclusions obtained with HSECs may be relevant to responses of other cell types during a systemic anthrax. Similar effect of T lymphocyte AKT phosphorylation was reported in LeTx-injected mice (Comer *et al.*, 2005a). Bommhardt *et al.* (2004) reported a correlation between decreased level of pAKT in lymphocytes and survival of septic mice. Our transcriptional analysis in the lung and spleen of Sterne spore-challenged mice also showed a strong modulation of the AKT canonical pathway genes (C. Bradburne, pers. commun.).

We found that reduced AKT phosphorylation in HSAECs was caused mainly by anthrax toxins secreted by *B. anthracis* into culture medium through a mechanism, which controls the calcium-dependent interaction of EC upon initiation of cell–cell contacts; this interaction normally induces a transient increase in AKT phosphorylation followed by the maturation of adherens junctions. Therefore, anthrax toxins are expected to interfere with the processes of epithelial development and repair after injury (Onder *et al.*, 2008). LeTx is known to cause apoptosis by inhibition of p38 in macrophages (Park *et al.*, 2002), and interference of LeTx and EdTx with the process of EC-mediated cell–cell signaling may represent an additional mechanism contributing to apoptosis of epithelium through induction of anoikis (Frisch & Screaton, 2001). The results obtained with inhibitors of ERK1/2 and p38 favor the hypothesis that inhibition of AKT phosphorylation takes place through the ERK1/2-PI3K pathway, as has been previously suggested (Pece *et al.*, 1999). However, the proapoptotic inhibition of p38 by LeTx seems to occur downstream of AKT activation (Laprise *et al.*, 2002).

AKT activates survival-related gene expression by transcription factors NF-κB and CREB and by apoptosis regulatory molecules, including BAD and mTOR pathway components (Franke *et al.*, 2003). CREB was previously implicated in anthrax pathogenesis. It is a major nuclear transcription factor important for cell survival, and it is positioned at the intersection of PI3K, MAPK and PKA signaling pathways as it can be activated via AKT, MEK/MAPK/p90RSK, or cAMP (Franke *et al.*, 2003; Kato *et al.*, 2007). It transduces cAMP activation of gene transcription and participates in a plethora of processes such as glucose metabolism, neuroendocrine control, innate immunity, and apoptosis. CREB can be inhibited by LeTx and activated by EdTx in macrophages (Park *et al.*, 2007). The upregulation of CREB observed in our experiments with both bacterial strains indicates that signals from pXO1-relevant factors do not play a predominant role in its regulation in HSAECs.

BAD phosphorylation takes place through the same major pathways as CREB (Datta *et al.*, 1999; Brazil *et al.*, 2002; Franke *et al.*, 2003; Tommasini *et al.*, 2008) and therefore is expected to be modulated by LeTx and EdTx. Increase of BAD phosphorylation at its major regulatory site S112 by the Sterne strain might reflect a predominant antiapoptotic effect of EdTx relative to the proapoptotic one of LeTx (Park *et al.*, 2007).

AKT is able to directly phosphorylate a constitutively expressed regulator of cellular glucose metabolism, GSK-3β. Inhibition of GSK-3β by AKT-mediated phosphorylation takes place in response to different external prosurvival stimuli (Eldar-Finkelman, 2002). We found a reduced GSK-3β phosphorylation by the Sterne infection in comparison with dSterne. In agreement with our data, it has been previously found that LeTx caused a GSK-3β-mediated cell cycle arrest in THP cells, which adaptively adjusted to LeTx and overrode the cycle arrest by activating the PI3K signaling pathway (Ha *et al.*, 2007). In contrast to GSK-3β, phosphorylation of its isoform, GSK-3α was strongly increased. The pathway responsible for this effect requires further investigation and may contribute to the perturbations of the glucose levels previously documented in anthrax infection (Slein & Logan, 1960; Eckert & Bonventre, 1963).

The optimal activation of the PI3K and the MAPK pathways depends on JAK/STAT signaling following binding of cytokine/interferon receptors with their ligands (Franke *et al.*, 2003). In connection with these observations, our data indicate that reduced phosphorylation of STAT3 is a plausible contributor to the pathogenicity of the toxigenic infection. The relationship between these processes is a subject of our ongoing investigation.

We conclude that interference with the host cell PI3K/AKT response represents an important part of a multifaceted anthrax pathogenic strategy. Kinomic analysis of host cell signaling provides a new opportunity to dissect host–pathogen interactions for possible therapeutic intervention candidates and mechanistic elucidation. Future studies will include experiments using specific *B. anthracis* gene knockouts in order to determine more precisely the bacterial factors involved in the host signaling response.

## Supporting Information

**Fig. S1.** Growth curves of Sterne and dSterne strains in the conditions of microarray experiments.

**Fig. S2.** Densitometry of Western blot data for AKT and GSK-α/β shown in Fig. 1.

**Table S1.** Proteins whose phosphorylation was significantly changed (*P* < 0.05) in HSAECs in response to *B. anthracis* challenge.
