# Supplementary figures and images for: Anthrax infection inhibits the AKT signaling involved in the E-cadherin-mediated adhesion of lung epithelial cells
Source: FEMS Immunol Med Microbiol. 2009 Jul;56(2):129–42. doi: 10.1111/j.1574-695X.2009.00558.x (PMC2734923; doi:10.1111/j.1574-695X.2009.00558.x)

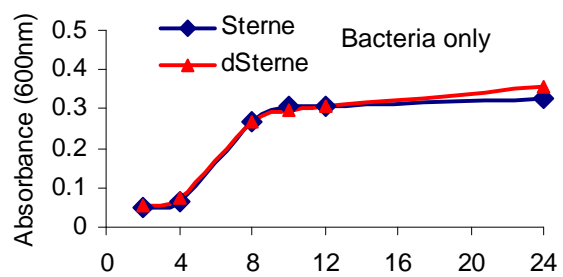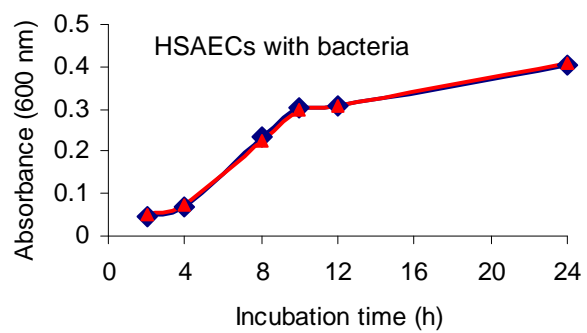

Supplement: Supplementary file 1 [file fim0056-0129-SD1.pdf]

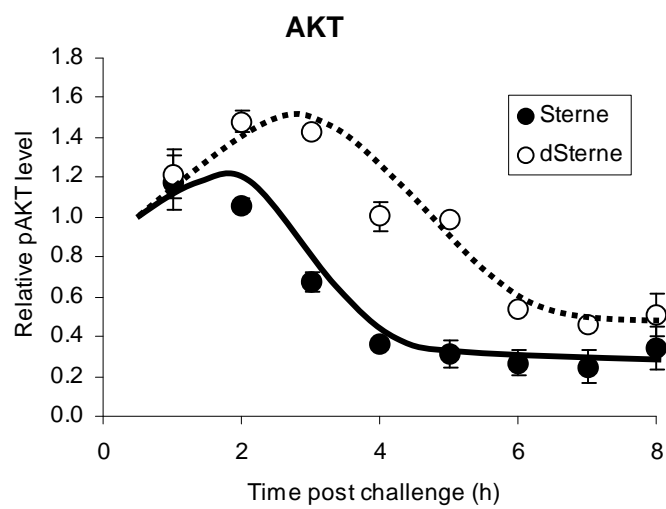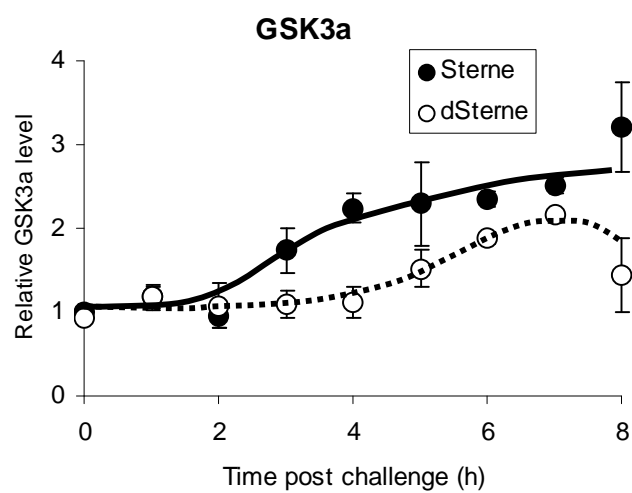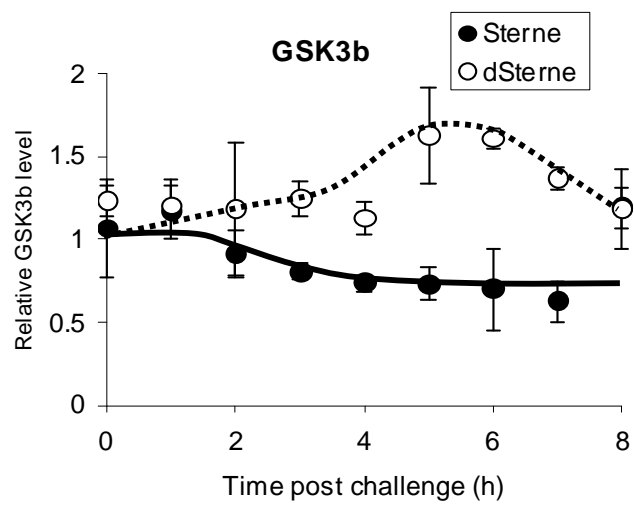

Supplement: Supplementary file 2 [file fim0056-0129-SD2.pdf]
