# Supplementary material for: Anthrax infection inhibits the AKT signaling involved in the E-cadherin-mediated adhesion of lung epithelial cells
Source: FEMS Immunol Med Microbiol. 2009 Jul;56(2):129–42. doi: 10.1111/j.1574-695X.2009.00558.x (PMC2734923; doi:10.1111/j.1574-695X.2009.00558.x)
Supplement: Supplementary file 3 [file fim0056-0129-SD3.doc]

Supplemental Table S1. Proteins which phosphorylation was significantly changed (p<0.05) in HSAECs in response to *B. anthracis* challenge

| **Name** | **Description** | **Function** | **Connectivity** |
| --- | --- | --- | --- |
| PKC | Protein kinase C | PKC family members are involved in a number of signal transduction processes including secretion, gene expression, proliferation and muscle contraction | 217 |
| ERK1/2 | Mitogen-activated protein kinase (MAPK) | Critical role in the regulation of cell growth and differentiation by a wide variety of extracellular signals including growth and neurotrophic factors, cytokines, hormones, and neurotransmitters. | 161 |
| AKT1-3 | V-akt oncogene murine thymoma homolog | Global control of the balance between survival and apoptosis. Inhibition of apoptosis through inactivation of BAD, Forkhead transcription factors and caspase-9. Regulation of glycogen synthesis through phosphorylation and inactivation of GSK-3. | 63 |
| STAT1-3 | Signal transducer and activator of transcription 1-3 | STATs are activated by a variety of cytokine and growth factor receptors, and cellular stress factors. | 46 |
| SAPK/  JNK1 | Mitogen-activated protein kinase 8 | SAPK/JNK is potently and preferentially activated by a variety of environmental stresses, including UV and gamma radiation, ceramides, inflammatory cytokines and some growth factors. | 28 |
| p38 | Mitogen-activated protein kinase (MAPK) | Control of cell responses by a variety of cellular stresses including osmotic shock, inflammatory cytokines, lipopolysaccharides (LPS), UV light, and growth factors. | 26 |
| CREB | Cyclic AMP response element binding protein | Transcription factor that activates target genes through cAMP response elements. CREB is activated by phosphorylation at S133 by various signaling pathways including ERK, Ca^2+^ and stress signaling. | 26 |
| eNOS | Endothelial nitric oxide synthase | Important enzyme in the cardiovascular system regulating blood pressure, vascular remodeling, and angiogenesis. Several protein kinases including AKT and PKA activate eNOS in response to various stimuli | 21 |
| JAK1/2 | Janus kinase 1 (a protein tyrosine kinase) | Members of the Janus family of tyrosine kinases are activated by a number of cytokine receptor ligands. | 18 |
| GSK-3β | Glycogen synthase  kinase 3 | Ubiquitously expressed serine/threonine protein kinase that phosphorylates and inactivates glycogen synthase. GSK-3β is a critical downstream element of the PI3 kinase/AKT cell survival pathway, and its activity can be inhibited by AKT-mediated phosphorylation. | 13 |
| mTOR | The mammalian target of rapamycin | Serine/threonine protein kinase, which plays a key role in cellular growth and homeostasis. mTOR transmits a positive signal to p70 S6 kinase and participates in the inactivation of the eIF4E inhibitor, 4E-BP1. mTOR is phosphorylated at S2448 via the PI3 kinase/AKT signaling pathway. | 13 |
| PYK2 | Protein tyrosine kinase also called CAKβ, RAFTK and CADTK | Nonreceptor tyrosine kinase is one of the signaling mediators for the MAPK important in cell spreading and migration. | 11 |
| BAD | BCL2-antagonist of cell death | AKT, p90RSK, PKA, and PKC promote cell survival via phosphorylation of BAD at S112, S136, and S155. | 4 |
| 4E-BP1 | Inhibitor of cap-dependent translation | Phosphorylation of 4E-BP1 in the PI3 kinase/AKT pathway activates translation. | 2 |
| p90RSK | Ribosomal protein S6 kinase, 90kDa, polypeptide 1 | Broadly expressed serine/threonine kinase activated by ERK in response to many growth factors, polypeptide hormones, and neurotransmitters. | 2 |
| FADD | Fas-associated death domain | An important adaptor in coupling death signaling from membrane receptors, such as the Fas ligand and TNF family, to caspase-8. | N/D |

* Number of network nodes in the human database of interactions (PathwayArchitect 2.0, Strategene, CA) for a particular protein
